# The mediating role of coping styles in the relationship between fear of COVID-19 and mental health problems: a cross-sectional study among nurses

**DOI:** 10.1186/s12889-024-17863-w

**Published:** 2024-02-21

**Authors:** Nurul Huda, Malissa Kay Shaw, Hsiu Ju Chang, Erwin, Suci Tuty Putri, Satriya Pranata

**Affiliations:** 1 Nursing Faculty, Universitas Riau, Pekanbaru, Riau Indonesia; 2 College of Arts and Sciences, University of Health Sciences and Pharmacy in St. Louis, St. Louis, MO United States; 3 School of Nursing, Department of Nursing, National Yang Ming Chiao Tung University, Taipei, Taiwan; 4 Department of Nursing, Faculty of Sport and Health Education, Universitas Pendidikan Indonesia, Bandung, Indonesia; 5 Faculty of Nursing and Health Sciences, Universitas Muhammadiyah Semarang, Semarang, Central Java Indonesia

**Keywords:** Fear of COVID-19, Coping, Mental health, Mediating, Nurse

## Abstract

Fear of being infected by coronavirus disease 2019 (COVID-19) could trigger mental health problems among nurses at the frontline. In such a situation, coping strategies are needed to deal with the imminent threat. The purpose of this study was to test the mediating effects of coping on relationships of fear of COVID-19 with anxiety, depression and post-traumatic syndrome among nurses who were in contact with COVID-19 patients. A cross-sectional and correlational research design was used to recruit a sample of 278 nurses who treated COVID-19 patients in four government referral hospitals in Indonesia. A bootstrap resampling procedure was used to test the significance of the total and specific indirect effects of coping on relationships of Fear of COVID-19 with anxiety, depression and post-traumatic syndrome. The nurses reported moderate levels of fear of COVID-19, considerable anxiety and depression, and a moderate level of coping. We found coping to be significantly negatively correlated with the reported levels of anxiety, depression and post-traumatic syndrome (*p* < 0.001). Coping mediated relationships of fear of COVID-19 on depression, anxiety and post-traumatic syndrome after controlling for relevant confounders for each dependent variable. This shows that enacting coping mechanisms is important to achieve an adaptive effect on nurses' mental health. Proper assessments and interventions should be tailored and implemented for nurses who have contact with COVID-19 patients to facilitate their use of coping strategies when needed in stressful situations.

## Introduction

The coronavirus disease 2019 (COVID-19) have been known as the biggest health challenge and crisis the world has experienced. Nurses as frontline healthcare staff have been one of the most vulnerable groups to contracting COVID-19 since they have been constantly in contact with the COVID-19 threat. In this situation, nurses play an important role in dealing with COVID-19 cases and subsequent complications which commonly require hospitalization [1, 2]. In Indonesia, there were 4,272,421 infected cases of COVID-19 in 2022, and the probability of transferring the infection to healthcare personnel was quite high [3]. Fear of being infected could trigger mental health problems among healthcare staff, especially nurses [4, 5].

Previous research found that during this pandemic period, the prevalence of mental health problems increased significantly among adults to 2.78% [6]. However, mental health problems are not only suffered by the general population but also by nurses who are in charge of the frontline among healthcare workers [7]. During this pandemic, the nurse’s working conditions were often harsh. In addition to being overworked, they were also at higher risk of contracting COVID-19, putting their lives and their families at risk while they fulfilled their duties [1, 8]. These conditions have contributed to the mental health problems experienced by nurses [1]. They have had to deal with the fear of contracting COVID-19, anxiety, depression, post traumatic syndrome (PTSD) and other emotional states stimulated by directly observing patients’ suffering and death [1, 9]. In addition, experiences of uncertainty and stigmatization have made many nurses reluctant to work and contemplate resignation [9]. Hence, it is not surprising that the levels of stress, anxiety, PTSD and depression symptoms among nurses were higher compared to other healthcare professionals which could have long-term psychological consequences [10].

Distress is acknowledged when stressful events have exceeded a person’s limited resources and may endanger their well-being. In such a situation, coping strategies are needed to deal with the imminent threat [11]. Coping involves an effort to manage adaptation demands and associated emotions [12]. During this pandemic, the use of certain coping strategies has been related to multiple outcomes including symptoms of depression and anxiety [13, 14]. A previous study found that coping was briefly proven to be a mediator of the relationship between nurses’ stressors and psychological distress [15–17]. However, to our best knowledge, the mediating role that coping plays in the relationships of fear of COVID-19 with anxiety and depression among nurses has never been studied.

Mental health problems among nurses have been proven to be linked to negative outcomes including physical and psychological problems which may deteriorate the quality of patients’ care [9]. In addition, hospitals effectiveness and productivity may be reduced due to staff’s intentions to leave [1]. Therefore, it is essential to study nurses’ mental health problems during this pandemic and explore the factors determining the negative impact on nurses’ mental health. This study aimed to analyze the mental health problems among nurses, including fear of COVID-19, depression, and anxiety, identify the determinants of mental health problems as well as the mediating role of coping strategies.

## Method

### Design, setting and population

A cross-sectional and correlational research design was used in this study. The patient inclusion criteria were nurses who had contact with COVID-19 patients, and the ability to speak the Indonesian language and fill out questionnaires. The exclusion criteria for this study were having been diagnosed with a major psychiatric morbidity, such as schizophrenia, or having a severe medical condition. Potential participants were recruited from inpatient wards, emergency wards and intensive care wards of 4 general government hospital in Indonesia. Data were collected between May and October 2022. The sample was composed of 278 nurses. Sampling and data collection were carried out face-to-face.

### Data collection and instruments

#### Sociodemographic and clinical characteristic information

Information concerning gender, age, marital status, religion, level of education, type of ward, and employment status were collected from participants.

#### Fear of COVID-19 scale

Participants’ fear of COVID-19 was assessed using the Fear of COVID-19 scale (FCV-19). The FCV-19S questionnaire consists of 7 items and scores range from 1 (strongly disagree) to 5 (agree). The total score, ranging between 7 and 35, is determined by adding up all item scores. The higher scores indicate a greater fear of COVID-19 and the lower scores designate a minor fear of COVID-19. Previous research found that this scale has solid psychometric properties, including high internal consistency with a Cronbach alpha value of 0.82 [18]. The Indonesian version of the FCV-19 scale showed good internal consistency with the value of Cronbach’s alpha being 0.87 [19].

#### Depression, Anxiety, and Stress Scale (DASS)

The DASS is comprised of three subscales of DASS-Stress, DASS-Anxiety and DASS Depression to, respectively, investigate stress, anxiety, and depression experienced 1 week before the survey [19]. Each subscale contains of 14 questions with a Likert scale from 0 to 3: 0, does not apply to me at all; 1, applies to me to some degree or some of the time; 2, applies to me to a considerable degree or a good part of the time; and 3, applies to me very much or most of the time. Scores for depression, anxiety and stress are calculated by summing the scores for the relevant items of each subscale. A higher score indicates a higher severity of each subscale. Cutoff points for the presence of indicators of stress, anxiety and depression are 14, 7 and 10, respectively. The DASS Indonesia version shows good reliability with α = 0.95 [19].

#### Brief Coping Orientation to Problems Experienced Inventory (Brief COPE)

The Brief COPE is the most commonly used measure to identify the nature of coping strategies implemented by individuals and explores 14 coping strategies. Every strategy has two items. It contains 28 items and is rated on a four-point Likert scale. In this study, Brief Cope consists of 14 coping strategies including acceptance, religion, planning, humour, positive reframing, instrumental support, active coping and emotional support, self-distraction, venting, self-blame, behavioural disengagement, denial and substance use [20]. A higher score represents greater coping strategies used by respondents. The Indonesian version of the Brief COPE exhibites good reliability with α = 0.825 [21].

#### *The Impact of Event Scale-Revised* (IES-R)

The Impact of Event Scale-Revised (IES-R) is one of the most commonly used metrics of post-traumatic stress disorder symptomatology. The IES-R questionnaire was validated by a previous study. The Indonesian IES-R revealed a Cronbach's alpha of 0.90 for the test and 0.92 for the retest for the total score [22]. The IES-R consists of 22 questions, each of which can receive a minimum score of 0 or a maximum score of 4. On a five-point Likert scale, the respondents indicate how often they experienced each symptom over the previous 7 days, ranging from 0 (not at all) to 4 (very much). The overall score is between 0 and 88 [23]. According to Horowitz et al. (1979) [24], a score of > 24 suggests some PTSD symptoms, > 33 is the optimal cutoff threshold, and > 37 indicates immune system suppression and severe PTSD.

### Data analysis

The collected data were managed and analyzed by IBM SPSS Statistics for Windows version 21 (IBM Corporation, Armonk, NY, USA). Descriptive statistics and frequency distribution were performed to analyse the participants' demographics and clinical characteristics. The Kolmogorov–Smirnov method was used to test for normal distribution. As the assumption of normality was rejected, the parametric tests were then used to analyze the data. The Mann–Whitney U and Kruskal–Wallis U tests were used to explore determinant factors [25], particularly demography factors, which influence anxiety, depression and post traumatic syndrome among nurses rather than correlations. This resulted in the identification of the relevant covariates that were included in the mediation analysis. All statistical tests were 2-sided with the level of significance set at 0.05.

The PROCESS Macro for SPSS version. 4.2 was used for testing the mediating effects of coping. We chose 10,000 bootstrap samples as recommended by Hayes [26]. The bootstrapping method is preferred since it can provide accurate indirect effects of coefficients for the asymmetric distribution of the data as found in the present study [26, 27]. Furthermore, to control type 1 errors, this method can own higher statistical power [26]. Fear of COVID-19 was included as an independent variable. Copings functioned as a mediator. Anxiety and depression were analyzed as dependent variables. The indirect effect test was conducted to confirm the mediating effect. The indirect effect is statistically significant at 0.05 if the 95% confidence interval (CI) does not encompass zero. Mediation was considered successful if the indirect effect was at a significant level. On this occasion, the direct effect remained significant (partial mediation) or may have been diminished (complete mediation). A higher indirect effect score indicates the more important mediator [26].

### Ethical considerations

This study was approved by the Ethical Committee Board of the Faculty of Nursing at the University of Riau in Indonesia (IRB No: 430/ UN.19.5.1.8/KEPK.FKp/2022). Informed consent to participate in this study was obtained from all patients.

## Results

### Response rate

Out of the 350 participants that we approached to participate in this study, 330 of them willing participated. The reasons for refusal to participate included limited time or no particular reason offered. 52 questionnaires were excluded from the data analysis because of incomplete data. In total, 278 questionnaires were used for the final data analysis, resulting in a final response rate of 84.2%. By using a mediated analysis with a medium effect size, this sample size fulfilled the minimal sample size and achieved a power of 0.8.

### Socio-demographic characteristics

As shown in Table 1, respondents were primarily female (70.9%), married (74.1%), Muslim (94.6%), and had graduated with a bachelor of nursing degree (53.2%). The mean age of respondents was 32.69 (SD = 6.0) years. Regarding their employment status, 58.3% of the participants were volunteers. Regarding the type of ward they work in 58.3%, 27.3% and 14.4% of the respondents were in charge of the inward unit, emergency unit and intensive care unit, respectively. The mean working time length since the first visit to the hospital was 6.71 (SD = 5.31). The mean score of fear of COVID-19 was 20.26 (SD = 6.00). Based on the categorical data 7.6%, 11.5% and 17.7% of participants had depression, post-traumatic syndrome disorder and anxiety symptoms, respectively.

**Table 1:** Sociodemographic and clinical characteristics

Factor	**Respondent (*N* = 2780) Frequency (%)**
Mean of age ± SD	43.96 ± 6.00
**Mean of working time**	6.71 ± 5.31
**Fear of COVID-19**	20.26 ± 6.00
Gender	
• Male	71 (29.1%)
• Female	197 (70.9%)
Marital Status	
• Married	206 (74.1%)
• Single	71 (25.9%)
Working Ward	
• Emergency	76 (27.3%)
• Inward care unit	162 (58.3%)
• Intensive care unit	40 (14.4%)
Religion	
• Muslim	263 (94.6%)
• Non-Muslim	15 (5.4%)
Education Background	
• Diploma	130 (46.8%)
• Bachelor	148 (53.2%)
Employment Status	
• Civil servant	76 (27.3%)
• Honorary	48 (14.4%)
• Volunteer	162 (58.3%)
• Upper and Similar to Indonesia's minimum wage	338 (76.8%)
Depression	
• Normal	246 (88.5%)
• Mild depression	11 (4.0%)
• Moderate depression	16 (5.8%)
• Severe depression	3 (1.1%)
• Very severe depression	2 (0.7%)
Anxiety	
• Normal	21 (77.6%)
• Mild anxiety	17 (6.6%)
• Moderate anxiety	31 (11.2%)
• Severe anxiety	9 (4.7%)
• Very severe anxiety	5 (1.8%)
Post Traumatic Syndrome	
• PTSD	32 (11.5%)
• No PTSD	246 (88.5%)
Fear of COVID-19	
• Fear	135 (48.6%)
• No fear	143 (57.4%)

*SD* standard deviation

### Differences in socio-demographic and clinical characteristics regarding depression, anxiety and QOL

Results indicated that depression was associated with employment status (x^2^ = 13,458, *p* = 0.001). Anxiety was associated with marital status and employment status (x^2^ = 2.547, *p* = 0.011 and x^2^ = 14.242, *p* = 0.001, respectively). Furthermore, the post-traumatic syndrome was related to the type of ward and employment status (x^2^ = 7.57, *p* = 0.023 and x^2^ = 13, 19, *p* = 0.001, respectively). All of these associated factors were used as confounders in the subsequent mediation analyses.

### Coping as a mediator

A regression analysis was used to test the hypothesis that coping mediated the effects of fear of COVID-19 on depression and anxiety after controlling employment status for depression and marital and employment status for anxiety.

**Fig. 1:**
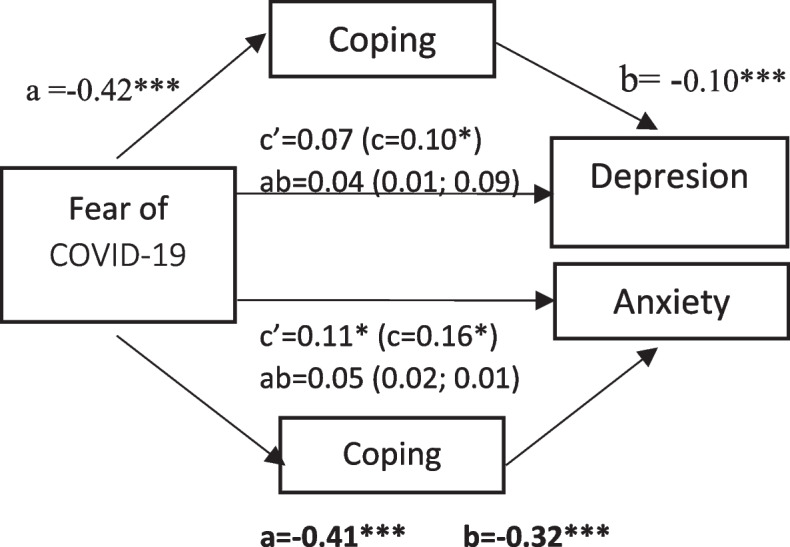
Mediating effects of coping on relationships of fear of COVID-19 with depression and anxiety. Regression coefficients (a, b, c, c’) are standardized with standard errors in parentheses. The 95% confidence intervals are displayed in brackets. c = total effect; c’ = direct effect; ab = indirect effect. Confounders for depression include employment status and confounders for anxiety include employment status and marital status

Results showed that after controlling for relevant confounders for each dependent variable, fear of COVID-19 had significant effects on depression, with coping having an indirect effect among them (Fig. 1). However, the relationship between fear of COVID-19 and depression noticeably decreased but did not show significant results after controlling for coping. These results indicated that coping fully mediated the relationship between fear of COVID-19 and depression. In addition, coping was also a significant mediator in the association of fear of COVID-19 with anxiety (Fig. 1). The relationship between fear of COVID-19 and anxiety showed depletion but still proved significant results which underpinned the hypothesis of partial mediation.

**Fig. 2:**
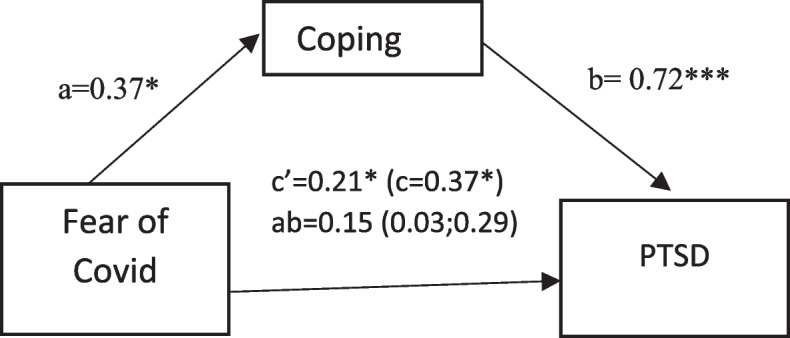
Mediating effects of coping on relationships of fear of Covid with PTSD. Regression coefficients (a, b, c, c’) are standardized with standard errors in parentheses. The 95% confidence intervals are displayed in brackets. c = total effect; c’ = direct effect; ab = indirect effect. Confounders for PTSD include the type of ward and employment status

Figure 2 presents the results of the analysis regarding coping as a mediator between fear of Covid 19 and post-traumatic stress disorder. Results indicated that fear of Covid 19 was a significant predictor of coping (B = 0,37, *p* < 0.01) and coping was a significant predictor for PTSD (B = 0,72, *p* < 0,001). Fear of COVID was also a significant predictor of PTSD (B = 0.37, *p* < 0.01). Moreover, after controlling for relevant confounders for each dependent variable, the results revealed that coping exerted an indirect effect on the dependent variables. These relationships decreased but remained significant thus supporting the hypothesis regarding partial mediation. These results indicated that coping partially mediated the relationship between fear of Covid-19 and PTSD. Moreover, from the statistical results, it can be concluded that only patients with PTSD experience mental health problems. If the PTSD variable is removed, fear of Covid and mental health problems will no longer be related. These results show that PTSD plays a major role in the incidence of mental health problems and fear of COVID. Coping is not proven to be a mediator between fear of Covid 19 on depression and anxiety if PTSD variables are excluded.

## Discussion

The major purpose of this study was to test the mediating effects of coping strategies on the relationships between fear of COVID-19 and emotional symptoms (anxiety and depression) and post-traumatic syndrome disorder after controlling for relevant confounding factors. We found that coping fully mediated the relationships of fear of COVID-19 with depression. In addition, coping also partially mediated the relationship between fear of COVID-19 and anxiety and also the relationship between fear of COVID-19 to post-traumatic syndrome disorder. Fear of COVID-19 was the most prevalent mental health problem among nurses, followed by anxiety, post-traumatic syndrome disorder and depression. Previous studies in similar situations such as the SARS pandemic also found fear of infection was a common problem and was one of the main stressors among nurses [1, 18]. In addition, similar research identified that fear of COVID-19 was a significant predictor of depression and anxiety in nurses, accounting for 34% of variance in depression and 62% in anxiety [28, 29].

The results of this study also found that frontline nurses were vulnerable to PTSD. Only patients with PTSD experienced mental health problems. If the PTSD variable is removed, fear of Covid and mental health problems will no longer be related. These results show that PTSD plays a major role in the incidence of mental health problems and fear of COVID. PTSD is an anxiety disorder that makes sufferers remember traumatic occurrences. Nurses are probably more vulnerable to PTSD due to closer and more frequent contact with COVID-19 [30]. These results are in line with a previous study that found more than 4 in every 10 nurses screened positive for higher levels of PTSD during the COVID-19 pandemic [31]. Previous studies also found that half of frontline nurses experienced PTSD and approximately 41% of them had severe emotional fatigue [31, 32]. This could explain why one to two years after the infection outbreaks, nurses still experienced higher levels of mental health problems and PTSD. Constantly feeling afraid and haunted by traumatic occurrences might trigger depression. Therefore, immediate interventions to protect nurses against traumatic events of this pandemic should be undertaken [31].

As expected, coping was a mediator between fear of COVID-19 and depression, anxiety and PTSD. Less use of coping strategies could increase depression, anxiety and PTSD [33]. During the COVID-19 pandemic, a sudden rise in the number of critically ill patients and the burden of decision-making affected stress and anxiety. Some studies have reported the incidence of PTSD following a sense of uncertainty and fear of disease transmission to oneself and others [31, 34]. These findings are similar to previous research which found that coping was a significant mediator among stressors such as fear of COVID-19 on depression and anxiety in a sample of nurses [9]. Coping is the individual continuous effort to adapt one’s behaviour and cognition to cope with stressful demands beyond one’s resources [35]. Individual coping styles are vital for influencing individual stress levels which can directly affect the strength of response to the stressor [33]. When faced with stress, individuals with appropriate coping strategies have positive thoughts and solutions (e.g. constructive actions). Therefore, developing appropriate coping strategies for nurses in the context of the COVID‐19 pandemic could be a crucial factor for nurses' mental health and reduce the risk of PTSD. Hence, Nurses should be provided with information regarding coping skills and treatment for anxiety, depression, PTSD and other mental health disorders.

Nurses were at a high risk of stress-related mental illness during this pandemic since they were the key frontline healthcare workers in hospitals. Anxiety and depression are the most common psychological problems encountered among nurses who were in contact with COVID-19 [1, 36]. The unprecedented nature of the disease and the continuous growth in the number of cases and deaths are likely to continue to cause depression and anxiety among nurses. Additionally, the prolonged fear of infecting their family and children increases healthcare workers’ stress and leads to depression and anxiety [28, 37, 38]. An appropriate coping strategy makes people more skilled in coping with such pressures in life and work, which subsequently reduces anxiety and depression. Although nurses felt that COVID-19 is a roller-coaster transitional journey, the use of various coping strategies has been found to effectively reduce their depression and anxiety [5]. The results of this study further support coping styles representing a potential predictive factor of depression and anxiety among nurses who were in contact with COVID-19 patients. With the implementation of appropriate coping skills and therapeutic interventions, nurses will be able to let go of the negative impacts that the COVID-19 pandemic has caused and reintegrate into their roles as caring and entrusted healthcare providers [9]. Additionally, social support plays an important role in reducing anxiety and depression, especially among nurses [39, 40]. Moreover, the power of spirituality in conjuncture with social support may reinforces someone's coping when facing problems [40, 41]. Spirituality can produce more sincerity about one’s problems and make it easier to accept the challenges they face in life [41, 42]. Integrating social support and spirituality into coping strategies will assist in the reduction of stress and depression [41].

In this study, we found that almost half of the nurses feel fear of COVID-19. These findings are similar to two previous studies which assessed the prevalence of COVID-19 fear using a similar instrument to the Fear of COVID-19 Scale [18]. Previous research estimated that 45.2% of nurses feared COVID-19 while 18.1% had a particularly strong fear associated with COVID-19 [18]. However, no cutoff points were mentioned for these prevalence estimates. During the COVID-19 pandemic, the fear experienced was about either being infected or infecting others, a fear that greatly impacted frontline healthcare workers, such as nurses. Longstanding fear may stimulate the behavioural immune system, which usually leads to aversive emotions, cognition and behavioural responses [4, 28]. In addition, prolonged exposure to fear may lead to emotional and distress-related disorders [4]. Hence, as fear may help in explaining several of these consequences, it is important to understand what creates this fear and what the predictors are.

The findings from our study have significant implications. In clinical situations, hospital management must regularly assess nurses’ coping styles and support them in choosing and practising appropriate cope. Such assessment may identify a need for support and assistance in coping more readily. To cope psychologically with the mental health problems of the COVID-19 epidemic, nurses must create new ways of coping or change their preexisting routines. In addition, it is important for a public mental health response to address PTSD, especially post-pandemic and even longer term. Early detection of PTSD among vulnerable populations, including nurses, is important to improve post-pandemic mental health and recovery. Our findings also point to the importance of developing interventions to increase nurses’s coping abilities with hospital management’s support. This kind of intervention is believed to directly reduce the mental health problems among nurses and reduce PTSD [1, 43]. With the implementation of healthy coping skills and therapeutic interventions, nurses will soon be able to let go of the negative impacts the COVID-19 pandemic has caused and reintegrate into their roles as caring and entrusted healthcare professionals [9].

To the best of our knowledge, this is the first study which investigated the role of coping as a mediator in the relationships of fear of COVID-19 with depression, PTSD and anxiety among nurses in Indonesia. The strength of this study is that we involved a large sample of nurses from 4 regions in Indonesia. However, this study has several limitations that should be recognized when generalizing the results. First, this study was only applied in the western part of Indonesia, and it is not possible to generalize the findings to all nurses in the eastern part of Indonesia. Second, this study design was cross-sectional, which prevented any identification of the direction of causation. Other similar studies need to be conducted in other countries for comparison.

## Conclusions

We found that coping was a mediator of the relationship of fear of COVID-19 with anxiety and depression. These results highlight the importance of coping among nurses who have had contact with COVID-19 patients. The hospital management system must address the regular assessment of coping strategies among nurses to detect the type of coping used. Appropriate interventions must be implemented to reduce depression and anxiety.

## Data Availability

Not applicable.
